# Peritoneal defect herniation causing small bowel obstruction: a rare complication of transabdominal preperitoneal repair

**DOI:** 10.1093/jscr/rjab263

**Published:** 2021-07-23

**Authors:** Enda Hannan, Olivia Baird, Meghan Feeney, Eoghan Condon

**Affiliations:** Department of Colorectal Surgery, University Hospital Limerick, Limerick, Ireland; Department of Colorectal Surgery, University Hospital Limerick, Limerick, Ireland; Department of Colorectal Surgery, University Hospital Limerick, Limerick, Ireland; Department of Colorectal Surgery, University Hospital Limerick, Limerick, Ireland

**Keywords:** laparoscopic, inguinal hernia, transabdominal preperitoneal, peritoneal defect herniation, small bowel obstruction

## Abstract

Laparoscopic approaches to inguinal hernia repair are becoming increasingly more popular as they offer many advantages to open techniques including faster recovery and lower rates of wound infection. However, it is important to recognize complications associated with newer techniques which only become apparent with increased volume and experience. In this report, we describe a rare case of small bowel obstruction (SBO) secondary to peritoneal defect herniation post-transabdominal preperitoneal repair (TAPP). This is an uncommon complication that is sparsely reported in the literature but may have devastating consequences for the patient if unrecognized or mistakenly attributed to adhesional SBO. A high index of suspicion for internal herniation and a low index for reoperation are important with SBO in the early postoperative phase post-TAPP.

## INTRODUCTION

Inguinal hernia (IH) repair is one of the most commonly performed surgical procedures, with more than 20 million cases annually worldwide [1]. Over the past two decades, there has been a paradigm shift from open to laparoscopic approaches, such as the transabdominal pre-peritoneal repair (TAPP) [1]. This involves developing the pre-peritoneal space overlying the hernia and reducing the hernia sac, after which a mesh is placed in the pre-peritoneal space overlying the defect. The peritoneal flap is then secured covering the mesh by tackers or laparoscopic suturing [1]. TAPP is widely accepted as safe and feasible, with low morbidity and recurrence [1]. This approach allows the patient to benefit from the advantages of laparoscopic surgery, including less post-operative pain, improved cosmesis and an earlier discharge [1].

However, no operation is without risk, and it is important to recognize and document complications associated with newer techniques which may only become apparent with increased volume and experience. In this report, we describe a rare case of small bowel herniation through a peritoneal defect post-TAPP resulting in intestinal obstruction.

## CASE REPORT

A 75-year-old man presented to the emergency department 3 weeks post-TAPP for bilateral IH with a 2-day history of crampy abdominal pain, vomiting and constipation. His abdomen was soft but diffusely tender and grossly distended on examination, with abdominal X-ray showing dilated small bowel loops. Routine laboratory investigations were unremarkable. A wide bore nasogastric tube (NGT) was placed which immediately aspirated 1350 ml of bilious fluid. Computed tomography (CT) of the abdomen was performed which diagnosed small bowel obstruction (SBO) with a transition point in the right iliac fossa of unclear aetiology. Initially suspecting an adhesional SBO, a trial of conservative management was commenced. However, this was unsuccessful, with 1700-ml draining from the NGT over the next 24 hours. Given the lack of clinical improvement, surgical exploration was warranted. As the patient had been adequately decompressed by NGT, a laparoscopic approach was used.

**Figure 1:**
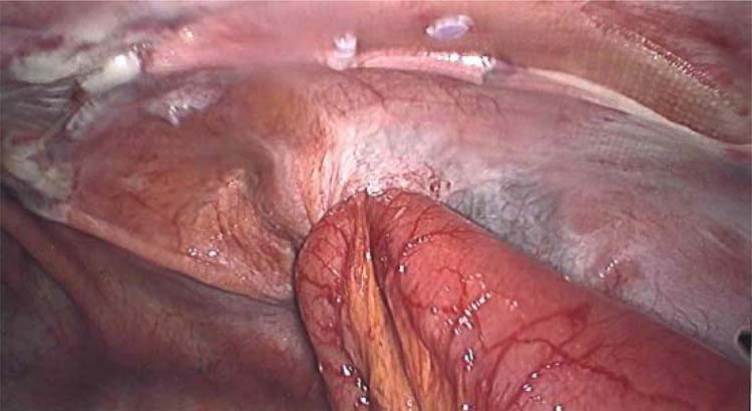
Small bowel herniation via peritoneal defect post TAPP.

**Figure 2:**
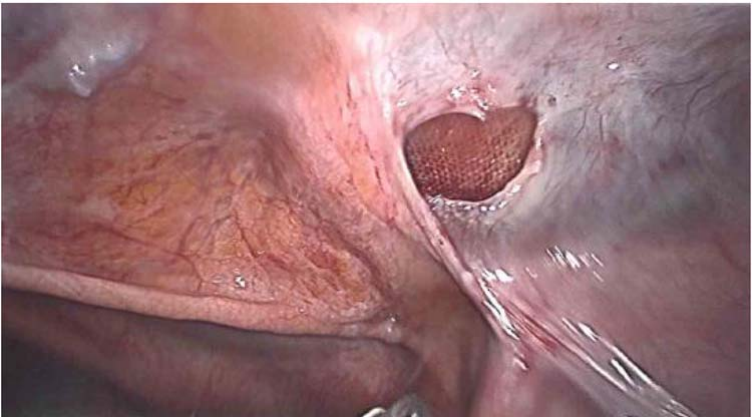
Peritoneal defect post-reduction of herniated small bowel with visible mesh.

**Figure 3:**
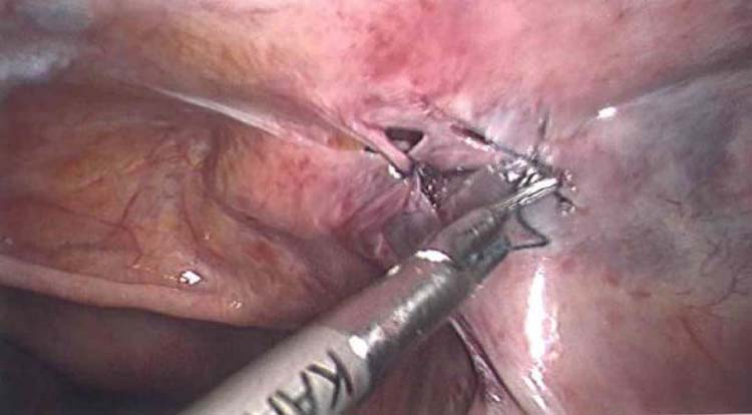
Laparoscopic suture closure of peritoneal defect.

Laparoscopy utilized the same access points as prior TAPP, a 10-mm infraumbilical port and two 5-mm ports in the left and right upper abdomen. This revealed that a loop of distal ileum had herniated into a small defect in the peritoneal flap overlying the right sided hernia repair site (Figs 1 and 2). The herniated small bowel was gently reduced and inspected, revealing it to be viable. The peritoneal defect was then closed laparoscopically by a 3.0 V-Loc™ barbed absorbable suture in a continuous manner (Fig. 3). The patient had an uneventful recovery and was discharged on the 5th post-operative day. He remains well at outpatient follow-up.

## DISCUSSION

The past two decades have seen an evolutionary change in the surgical management of IH, with the unambiguous advantages of minimally invasive surgery transforming a procedure traditionally associated with considerable postoperative pain and slow recovery [1]. Laparoscopic repair is recognized as the gold standard for bilateral and recurrent IH and is also accepted as a safe and feasible approach for unilateral IH [1]. Both the TAPP approach and total extraperitoneal approach are widely utilized, with TAPP preferred by many due to being performed with a more familiar intraperitoneal access and view [1]. However, with these newer approaches comes the potential for complications previously not associated IH repair. SBO secondary to peritoneal defect herniation (PDH) is a rare but serious complication of TAPP, with the potential for devastating consequences if unrecognized.

Less than 20 cases of PDH secondary to TAPP have been reported [2–5]. Kapiris *et al.* [2] report 7 cases of this in 3017 patients over 7 years (0.23%). Interestingly, they noted that the incidence decreased with sutured peritoneal closure compared with tacker closure. This is likely a result of less tension on the peritoneal flap, thus reducing the risk of tearing. However, suture closure does not eliminate this complication. Narayanan *et al.* [3] report two PDH in a case series of robotic TAPP with sutured peritoneal closure. In both cases, they observed that the peritoneum was too attenuated to allow closure of the peritoneal defect, and thus packed the defect with Surgicel® to obliterate the dead space and prevent reincarceration [3]. The authors suggested that the use of smaller bites, reducing pneumoperitoneum and using smaller suture needles may reduce the risk of peritoneal tear and subsequent PDH [3].

PDH is not exclusive to TAPP. A small number of cases of PDH secondary to laparoscopic trocar insertion have been reported with ports as small as 5 mm [4]. As with our case, such a rare occurrence can pose a diagnostic challenge, with potentially serious implications for a delayed diagnosis. It is tempting to consider SBO in a patient with previous abdominal surgery to be adhesional in nature, the vast majority of which will not require surgery. However, a high index of suspicion for internal herniation such as PDH should be maintained in the early post-operative phase following laparoscopic surgery. Adhesional SBO following a minimally invasive approach is much less frequent than following laparotomy, especially in the early post-surgery window. Cross-sectional imaging may be helpful in diagnosis; however, it may also mislead, as the thin layer of peritoneum responsible for SBO is unlikely to be visualized on CT, with a sharp transition point without clearly visualized pathology being mistakenly attributed to adhesion formation. For this reason, it is important to have a high index of suspicion and low threshold for re-operation for SBO in the early post-operative period, where a delay in intervention may result in bowel ischaemia, necrosis, perforation, peritonitis and mortality.

Our case highlights three important learning points. Firstly, SBO secondary to PDH post-TAPP is a highly uncommon complication that was not previously encountered in our high-volume practice and is sparsely reported in the literature. However, an awareness of this is important as TAPP becomes more frequent, as a delay in diagnosis can have serious consequences. Secondly, diagnosis is challenging, as CT appearances may mimic adhesional SBO; therefore, a high index of suspicion and low index to re-operate in this scenario are essential. Finally, this complication can be managed laparoscopically should the patient condition allow it, maintaining the post-operative benefits of minimally invasive surgery.
